# Frailty, Inflammaging, and Polypharmacy as Interacting Determinants of Falls in Older Women: A Clinical and Pharmacological Perspective in Primary Care Settings

**DOI:** 10.3390/diseases14100374

**Published:** 2026-10-12

**Authors:** Zvonimir Bosnić, Hrvoje Pitlović, Ivana Šofić, Maja Miletić, Danijel Marinčić, Katarina Šarčević Ivić-Hofman, Ivan Lekić, Karlo Miličić Juhas, Valerija Miličić, Tatjana Bačun

**Affiliations:** 1 Faculty of Medicine, Josip Juraj Strossmayer University of Osijek, Josipa Huttlera 4, 31000 Osijek, Croatia; zbosnic191@gmail.com (Z.B.); hrpitlovic@gmail.com (H.P.); tbacun@mefos.hr (T.B.); 2 General Hospital “Dr. Josip Bencević”, Andrije Štamapara 42, 35000 Slavonski Brod, Croatia; ivanasofic@yahoo.com (I.Š.); majam1013@gmail.com (M.M.); marincicdanijel@gmail.com (D.M.); 3 University of Slavonski Brod, 35000 Slavonski Brod, Croatia; ksihofman@unisb.hr; 4 Department of Internal Medicine and History of Medicine, Faculty of Medicine Osijek, Josipa Huttlera 4, 31000 Osijek, Croatia; ivlekic@mefos.hr; 5 Division of Internal Medicine, Health Center Zagreb Centar, Runjaninova 4, 10000 Zagreb, Croatia; 6 Clinical Hospital Center “Sestre Milosrdnice”, Vinogradska Cesta 29, 10000 Zagreb, Croatia; 7 Faculty of Medicine Zagreb, 10000 Zagreb, Croatia; karlomj03@gmail.com; 8 University Hospital for Infectious Diseases “Dr. Fran Mihaljević”, 10000 Zagreb, Croatia; 9 Department of Clinical Medicine, Faculty of Dental Medicine and Health Osijek, Crkvena Ulica 21, 31000 Osijek, Croatia; 10 Division of Endocrinology, Department of Internal Medicine, University Hospital Centre Osijek, Josipa Huttlera 4, 31000 Osijek, Croatia

**Keywords:** frailty, falls, inflammaging, polypharmacy, older women, sarcopenia

## Abstract

Background: Falls in older women are multifactorial events driven by the complex interaction of age-related loss of physiological reserve, frailty, sarcopenia, chronic low-grade inflammation, multimorbidity, and medication exposure. Older women represent a substantial proportion of fall-related injuries and fractures, yet available evidence is highly heterogeneous. Since many studies focus on mixed-sex cohorts, we must differentiate women-specific findings from general geriatric data. Methods: A structured narrative review was conducted to integrate biological, clinical, and pharmacological evidence on falls in older women, focusing particularly on primary care. The initial literature search covered PubMed, Scopus, Web of Science, and Embase from January 2000 to March 2025. To ensure the integration of recent high-level evidence, a supplementary search and backward citation tracking were subsequently executed through 31 August 2026, aligning with peer-review recommendations. Women-specific evidence was prioritized over mixed-sex cohorts. Given that the review aims for an interpretive synthesis rather than quantitative effect estimation, a meta-analysis and formal risk-of-bias assessments were omitted. Discussion: Frailty and sarcopenia are consistently associated with falls and functional decline in older adults. In contrast, inflammaging and metabolic dysregulation are more strongly supported as contributors to frailty, sarcopenia, and functional vulnerability than as independently established causal determinants of falls. Medication-related fall susceptibility may arise through several mechanisms, including sedation, impaired attention and psychomotor function, orthostatic hypotension, bradycardia, electrolyte and glucose disturbances, gait impairment, and adverse skeletal effects. Psychotropic agents, anticholinergic drugs, opioids, selected cardiovascular drugs, and other fall-risk-increasing drugs warrant particular attention in patients with reduced physiological reserve. Medication review and appropriate deprescribing should be considered components of multidomain fall-prevention strategies rather than stand-alone interventions. Conclusions: The principal contribution of this review is not the identification of new individual fall-risk factors, but the integration of established biological vulnerability, functional impairment, and pharmacological burden within a sex-sensitive primary-care framework. This approach may help clinicians consider frailty, medication exposure, and women-specific modifiers simultaneously during fall-risk assessment. The proposed framework is hypothesis-generating and should not be interpreted as a prospectively validated mechanistic or prediction model.

## 1. Introduction

Falls are among the leading causes of morbidity, disability, hospitalization, institutionalization, and mortality in older adults and represent a major public-health challenge worldwide [1,2]. Older women experience a substantial burden of fall-related injuries, particularly fractures, functional decline and loss of independence [3]. Beyond the immediate physical consequences falls may lead to fear of falling, reduced mobility, social isolation, diminished quality of life and increased healthcare utilization, imposing considerable burdens on patients, caregivers, and healthcare systems [1,3].

The distinction between susceptibility to falling and susceptibility to fall-related injury is particularly important in older women. Biological and functional factors such as frailty, sarcopenia, impaired balance, gait disturbances, cognitive impairment, and medication-related adverse effects may increase the probability of a fall, whereas osteoporosis and reduced bone strength primarily increase the likelihood that a fall will result in a fracture [4,5,6,7]. These pathways frequently coexist but should not be considered interchangeable [5,6].

Frailty is an important component of fall susceptibility in later life and is characterized by reduced physiological reserve and diminished resilience to internal and external stressors [7]. Frail individuals have an increased risk of falls, disability, hospitalization, and mortality, while prefrailty represents a potentially modifiable stage during which targeted intervention may help preserve function [7]. Sarcopenia, characterized by progressive loss of muscle strength, muscle mass, and physical performance, is closely related to frailty and contributes to impaired mobility, balance and postural control [4,6].

Older women may have additional biological and clinical vulnerabilities that modify these relationships. Following menopause, declining estrogen levels contribute to accelerated bone loss and may adversely affect skeletal muscle and musculoskeletal function [5,6]. Women also have a higher prevalence of frailty than men in many populations and generally live longer, thereby accumulating a greater lifetime burden of multimorbidity, disability, osteoporosis, and medication exposure [8,9]. However, not all evidence concerning frailty, polypharmacy, sarcopenia, and falls has been generated specifically in women. Much of the available literature derives from mixed-sex older populations, and women-specific conclusions therefore require careful distinction from findings extrapolated from general geriatric evidence.

Age-related physiological changes further modify pharmacokinetic and pharmacodynamic responses to medications and may increase susceptibility to medication-related adverse effects [10,11]. Sedation, impaired attention, dizziness, orthostatic hypotension, bradycardia, visual disturbance, cognitive impairment, electrolyte disturbances, and gait instability are among the pathways through which medication exposure may contribute to fall susceptibility [10,11,12]. The clinical relevance of these effects is likely to depend not only on the medication itself but also on the underlying physiological reserve of the individual. A medication-related perturbation that is tolerated by a robust older adult may have greater functional consequences in a person with frailty, sarcopenia, multimorbidity, or impaired postural control [7,12,13].

Chronic low-grade inflammation, commonly referred to as inflammaging, has also been implicated in biological aging and frailty [14,15]. Persistent inflammatory activity is associated with muscle catabolism, reduced physical performance, fatigue, and other features related to functional decline. However, the evidence directly linking inflammaging to incident falls is less established than the evidence linking inflammation with frailty and sarcopenia [14,15,16]. In this review, inflammaging is therefore considered primarily as a potential contributor to biological vulnerability rather than as an independently proven causal determinant of falls.

Multimorbidity adds further complexity because multiple chronic diseases may simultaneously impair mobility, balance, cognition, sensory function, and exercise capacity while also increasing the need for pharmacological treatment. Polypharmacy and exposure to fall-risk-increasing drugs (FRIDs) are common in older adults, and several medication classes—including psychotropic agents, anticholinergic drugs, opioids, and selected cardiovascular drugs—have been associated with increased fall risk [12,13]. Medication count alone, however, may not adequately represent pharmacological vulnerability. Drug class, dose, cumulative sedative or anticholinergic burden, drug–drug interactions, renal decline, orthostatic effects, metabolic adverse effects and the patient’s level of frailty may all influence clinical risk. The individual relationships among frailty, sarcopenia, multimorbidity, polypharmacy, FRIDs and falls are already well established. Accordingly, the purpose of this review is not to propose these associations as novel findings. Rather, its intended contribution is to integrate these established biological and pharmacological domains within a sex-sensitive framework relevant to older women and primary care.

The aim of this structured narrative review is therefore to synthesize evidence concerning the interplay among frailty, inflammaging, sarcopenia, multimorbidity, and pharmacological burden in relation to fall susceptibility in older women. Attention is given to distinguishing evidence demonstrated specifically in women from sex-stratified evidence and from findings derived from general older populations. Based on this synthesis, we propose a clinically oriented conceptual framework for primary care that links biological vulnerability with medication-related stressors while recognizing that the proposed interactions remain hypothesis-generating and require prospective validation.

## 2. Materials and Methods

### 2.1. Study Design

This study was conducted as a structured narrative review to examine the relationships among frailty, inflammaging, sarcopenia, multimorbidity, polypharmacy, and falls in older women with particular emphasis on their clinical relevance in primary care. A structured narrative approach was selected because the objective was to integrate evidence from heterogeneous biological, clinical, epidemiological, and pharmacological literature rather than to estimate a pooled treatment effect or association measure. The review was therefore designed as an interpretive evidence synthesis and not as a systematic review or meta-analysis.

Attention was given to distinguishing evidence derived specifically from older women from sex-stratified analyses and from evidence generated in mixed-sex older populations. Where women-specific data were unavailable, findings from broader geriatric populations were considered for their mechanistic or clinical relevance but were not interpreted as women-specific evidence.

### 2.2. Literature Search Strategy

The initial literature search was conducted in PubMed/MEDLINE, Scopus, Web of Science, and Embase and covered publications from January 2000 through March 2025. The search was structured around the principal concepts addressed in this review: frailty, prefrailty, sarcopenia, falls, fall risk, older women, older adults, inflammaging, chronic inflammation, multimorbidity, polypharmacy, fall-risk-increasing drugs, deprescribing, medication-related adverse effects and fall prevention. Search terms were combined using Boolean operators (AND/OR) and adapted to the indexing structure and syntax of each database. A representative PubMed search query included combinations of the following concepts: (“frailty” OR “pre-frailty” OR “prefrailty” OR “sarcopenia”) AND (“falls” OR “fall risk” OR “recurrent falls”) AND (“polypharmacy” OR “fall-risk-increasing drugs” OR “deprescribing” OR “medication burden”) AND (“older women” OR “older adults” OR “aged”).

Additional searches were undertaken for mechanistic domains that were not consistently captured by the combined core search, particularly inflammaging, chronic low-grade inflammation, metabolic dysregulation, pharmacokinetic and pharmacodynamic changes, anticholinergic burden, orthostatic hypotension, and medication-related adverse effects. To ensure the inclusion of the most recent evidence, a targeted literature update was performed to identify recently published data and update selected high-priority topics. This update included recent systematic reviews, meta-analyses, clinical guidelines, consensus statements and clinically relevant observational studies published after the original search period. The updated search extended the literature coverage through 31 August 2026.

Backward reference screening of relevant reviews, guidelines and eligible studies was also used to identify additional publications considered important for the conceptual synthesis. Because this review was designed as structural narrative synthesis rather than a formal systematic review, complete verbatim database-specific search strings were not prospectively achieved for all databases. The search strategy is therefore reported at the level of the principal concepts, databases, time periods and supplementary targeted searches used to inform the review.

### 2.3. Eligibility Criteria

Eligible publications were required to address one or more biological, functional, clinical or pharmacological determinants relevant to falls, fall susceptibility, frailty, sarcopenia or functional decline in older adults.

Women-specific studies were prioritized. Evidence was classified conceptually according to whether it was derived from: (1) studies conducted specifically in older women; (2) mixed-sex studies reporting sex-stratified results; or (3) mixed-sex older populations without sex-specific analysis. Evidence from the third category was used to inform general geriatric mechanisms and clinical practice but was not presented as direct evidence specific to older women. Priority was given to systematic reviews, meta-analyses, clinical practice guidelines, consensus statements, randomized controlled trials and high-quality observational studies.

Observational studies were considered particularly relevant when they included clearly defined populations, appropriate adjustment for major confounders, clinically meaningful measures of frailty, physical performance, medication exposure, or falls and sufficient methodological detail to permit interpretation of the findings.

Studies were excluded when they primarily involved individuals younger than 60 years, were conference abstracts without sufficient methodological detail, were not available in English, or lacked clear relevance to the biological, clinical, or pharmacological pathways considered in this review.

Because the review also addresses mechanisms indirectly related to falls, studies examining frailty, sarcopenia, inflammation, metabolic dysregulation, or medication-related vulnerability were considered when they provided biologically or clinically relevant evidence, even when falls were not the primary outcome. Such evidence is identified in the narrative synthesis as indirect rather than direct evidence of fall risk.

### 2.4. Study Selection

Titles and abstracts identified through the literature search were screened for relevance to the predefined domains of the review. Potentially relevant publications were subsequently assessed in full text.

Study selection focused on publications contributing directly to one or more of the following domains: frailty and prefrailty; sarcopenia and physical performance; inflammaging and biological vulnerability; multimorbidity; pharmacological burden and fall-risk-increasing drugs; medication optimization; sex-specific vulnerability; and fall-prevention strategies relevant to primary care. Where multiple publications addressed the same question, greater weight was given to systematic reviews, meta-analyses, international clinical guidelines, large prospective studies and more recent high-quality evidence. Screening and eligibility assessment were performed independently by two reviewers, with disagreements resolved through discussion and consensus.

Additional relevant publications were identified by screening reference lists of key reviews, guidelines, and eligible studies.

### 2.5. Evidence Synthesis

Evidence was synthesized narratively because of substantial heterogeneity in study populations, exposures, outcomes, study designs, and biological and pharmacological domains considered. No formal risk-of-bias tool or quantitative meta-analysis was applied. Consequently, the review does not provide pooled effect estimates or formal certainty-of-evidence ratings. Instead, evidence was interpreted according to study design, population characteristics, sample size, consistency with other studies, methodological strengths and limitations, directness of the evidence to falls, relevance to older women and primary care.

For interpretive purposes, particular distinction was made between evidence directly examining falls and evidence examining intermediate outcomes such as frailty, sarcopenia, inflammatory activity, muscle weakness, orthostatic hypotension, cognitive impairment, or medication-related adverse effects. Similarly, women-specific evidence was distinguished from findings derived from mixed-sex populations to avoid attributing general geriatric observations specifically to older women without supporting sex-specific data. The synthesis was organized around three interconnected domains:

(1) Biological vulnerability, including frailty, sarcopenia, inflammaging, metabolic dysregulation and multimorbidity.
(2) Pharmacological burden, including polypharmacy, FRIDs, cumulative sedative and anticholinergic exposure, and age-related pharmacokinetic and pharmacodynamic changes.
(3) Clinical integration, including risk assessment, medication optimization, multidomain intervention and follow-up in primary care.

The conceptual framework developed from this synthesis should therefore be regarded as an interpretive and hypothesis-generating model.

The principal studies and guidance documents informing the biological, pharmacological, and clinical domains of the narrative synthesis are summarized in Table 1. The table is intended to illustrate the evidentiary basis of the proposed framework rather than present an exhaustive list of all publications considered.

## 3. Frailty as the Biological Basis of Fall Susceptibility

Frailty is a multidimensional clinical condition characterized by reduced physiological reserve and diminished resilience to internal and external stressors [18,33,34,35]. Rather than representing a single disease entity, frailty reflects the cumulative effects of age-related changes across multiple physiological systems, including musculoskeletal decline, chronic inflammation, neuroendocrine dysregulation, multimorbidity, and functional impairment [18,34,35]. Although the frailty phenotype and deficit-accumulation approaches differ conceptually, both identify older individuals at increased risk of adverse outcomes, including disability, hospitalization, falls, and mortality [17,18,35].

Falls are particularly relevant in the context of frailty because reduced physiological reserve may compromise the ability to compensate for disturbances in gait, balance, muscle strength, reaction time, or postural control [18,36,37]. A systematic review and meta-analysis identified frailty as a predictor of future falls among community-dwelling older people [19]. Frailty should, however, be considered a marker of increased vulnerability rather than a single direct cause of falling, because fall events typically arise from interactions among multiple intrinsic, pharmacological, behavioral, and environmental factors [19,24].

### 3.1. Prefrailty as a Potentially Modifiable Stage

Attention should be given to prefrailty which represents an intermediate state between robustness and established frailty and may provide an important opportunity for early intervention. Frailty trajectories are dynamic and present transitions between frailty states, including transitions toward less severe states, that have been observed in longitudinal studies [38]. However, the term “reversible” should be used cautiously because improvement is not universal and depends on baseline health status, comorbidities, intervention characteristics, adherence, and social context [27,28,38]. Evidence from systematic reviews and international clinical practice guidelines supports multicomponent physical activity, particularly interventions incorporating progressive resistance, balance, and functional exercise, for older adults with frailty or prefrailty [27,28]. Exercise should therefore be regarded as the principal evidence-supported intervention for improving physical function and potentially slowing frailty progression rather than implying that prefrailty can routinely be reversed [27,28]. Nutritional management should be individualized. Adequate protein and energy intake are important for maintaining muscle mass and function, particularly in older adults with sarcopenia, weight loss, or undernutrition [28,39]. However routine protein supplementation should not be presented as universally indicated for every individual with prefrailty or frailty. Nutritional supplementation is most appropriate when nutritional assessment identifies inadequate intake, weight loss, undernutrition, or sarcopenia [28,39]. Similarly, vitamin D supplementation should not be regarded as a specific treatment for frailty or routinely recommended solely for fall prevention in all older women. Correction of vitamin D deficiency should instead be considered when deficiency is documented or when supplementation is otherwise clinically indicated [24,40]. These distinctions support an individualized, multidomain approach in which exercise, nutritional assessment, management of chronic diseases, and medication review are tailored to the patient’s functional and clinical status rather than applied uniformly.

### 3.2. Women-Specific Vulnerability

Frailty is particularly relevant to older women because women have a higher prevalence of frailty than men across many populations [8,9]. A systematic review and meta-analysis by Gordon et al. demonstrated a higher prevalence of frailty in women, although sex differences in frailty-related outcomes remain complex [8].

Several biological characteristics may modify the clinical consequences of frailty in older women. Menopause-related estrogen decline contributes to accelerated bone loss and may adversely influence skeletal muscle and musculoskeletal function [6,7,41,42]. Importantly, these pathways should be interpreted separately: sarcopenia, impaired muscle function, and balance disturbances may contribute to fall susceptibility, whereas osteoporosis and reduced bone strength primarily increase the probability that a fall will result in fracture [5,6,32,43].

Women also have greater longevity and may therefore accumulate a substantial lifetime burden of multimorbidity, disability, and medication exposure [8,9,44]. However, sex differences in polypharmacy and medication-related fall risk are heterogeneous across populations. Consequently, evidence derived from mixed-sex older populations should not automatically be interpreted as demonstrating greater pharmacological risk specifically in women.

Social and contextual factors, including living arrangements, physical activity, socioeconomic conditions, social support, and access to healthcare, may further influence functional decline and vulnerability to falls [44,45]. These factors should be considered potential modifiers rather than inherent biological characteristics of women because their distribution varies between populations and healthcare systems.

### 3.3. Frailty and Polypharmacy

Frailty and polypharmacy frequently coexist in clinical practice. A systematic review demonstrated a consistent association between frailty and polypharmacy in older adults, although heterogeneity among studies and the predominantly observational evidence limit causal interpretation [22]. Multimorbidity frequently necessitates complex pharmacological treatment, while age-related changes in renal function, body composition, drug metabolism and pharmacodynamic sensitivity may increase susceptibility to medication-related adverse effects [10,11,22,46]. Medication-related effects such as sedation, dizziness, cognitive impairment, orthostatic hypotension, electrolyte disturbances and gait instability may further compromise functional capacity and contribute to fall susceptibility [12,23,24,46]. The relationship between frailty and polypharmacy should therefore be regarded as complex and potentially bidirectional rather than as a definitively established synergistic mechanism [22,47]. Frailty may increase vulnerability to medication-related adverse effects whereas adverse drug events, prescribing complexity and treatment burden may contribute to functional decline. However much of the supporting evidence is observational and residual confounding by multimorbidity and disease severity remains important [22,47]. Medication count alone should therefore not be interpreted as an adequate measure of pharmacological fall risk. Clinical assessment should additionally consider potentially inappropriate medications, fall-risk-increasing drugs, cumulative sedative and anticholinergic burden, drug–drug interactions, renal function, orthostatic symptoms, and the patient’s overall physiological reserve [12,23,24,25,48]. Medication review should consequently be incorporated into comprehensive frailty and fall-risk assessment. Deprescribing or medication optimization should be individualized and integrated into multidomain management rather than considered an isolated intervention capable of reversing frailty or independently preventing falls [24,25,49].

## 4. Biological Pathways Underlying Fall Susceptibility

Frailty-related fall susceptibility reflects the convergence of multiple biological and functional processes that may impair mobility, balance, neuromuscular performance, and the capacity to compensate for physiological stressors [18,19,20,24]. These processes should not be interpreted as equally established direct causes of falls. Sarcopenia and impaired physical performance have relatively direct associations with fall susceptibility, whereas inflammaging and metabolic dysregulation are more strongly supported as contributors to frailty, muscle dysfunction, and reduced physiological reserve [14,15,16,21]. In older women, these pathways may be further modified by menopause-related musculoskeletal changes, osteoporosis, multimorbidity, and accumulated treatment burden. However, because much of the mechanistic evidence derives from mixed-sex older populations, women-specific interpretation should be made cautiously [22,47,48].

### 4.1. Sarcopenia and Neuromuscular Decline

Sarcopenia is characterized by progressive impairment in muscle strength, muscle quantity or quality, and physical performance and represents an important component of frailty and mobility decline [4,6]. Current consensus emphasizes low muscle strength as a key characteristic of probable sarcopenia with reduced muscle quantity or quality supporting diagnosis and impaired physical performance indicating greater severity [4]. Loss of lower-extremity strength and muscle power may compromise gait stability, postural control, stair negotiation and the capacity to generate rapid corrective responses following an unexpected perturbation. These functional consequences provide a plausible and clinically relevant pathway linking sarcopenia with increased fall susceptibility [20]. Importantly muscle strength and physical performance appear to be more clinically informative for mobility and fall-related vulnerability than muscle mass considered in isolation [4,6]. This distinction is relevant in primary care because simple functional measures such as gait speed, chair-stand performance, and grip strength may identify clinically meaningful impairment even when direct assessment of muscle mass is unavailable [4].

In older women, menopause-related hormonal changes may contribute to changes in body composition and musculoskeletal function, while osteoporosis frequently coexists with sarcopenia [5,6,31,32]. The coexistence of sarcopenia and osteoporosis may increase both the likelihood of functional instability and the severity of injury if a fall occurs. However, these two consequences should be distinguished: sarcopenia predominantly relates to mobility and fall susceptibility, whereas osteoporosis primarily modifies fracture risk following a fall [5,20,31,32]. Accordingly, sarcopenia should be regarded as an important component of the functional vulnerability underlying falls, while avoiding the assumption that all sarcopenia-related mechanisms have been demonstrated specifically in older women.

### 4.2. Inflammaging and Chronic Low-Grade Inflammation

Inflammaging refers to persistent, low-grade systemic inflammatory activity associated with aging and has been implicated in frailty, sarcopenia, cardiovascular disease, metabolic dysfunction, and functional decline [14,15,16].

**Figure 1:**
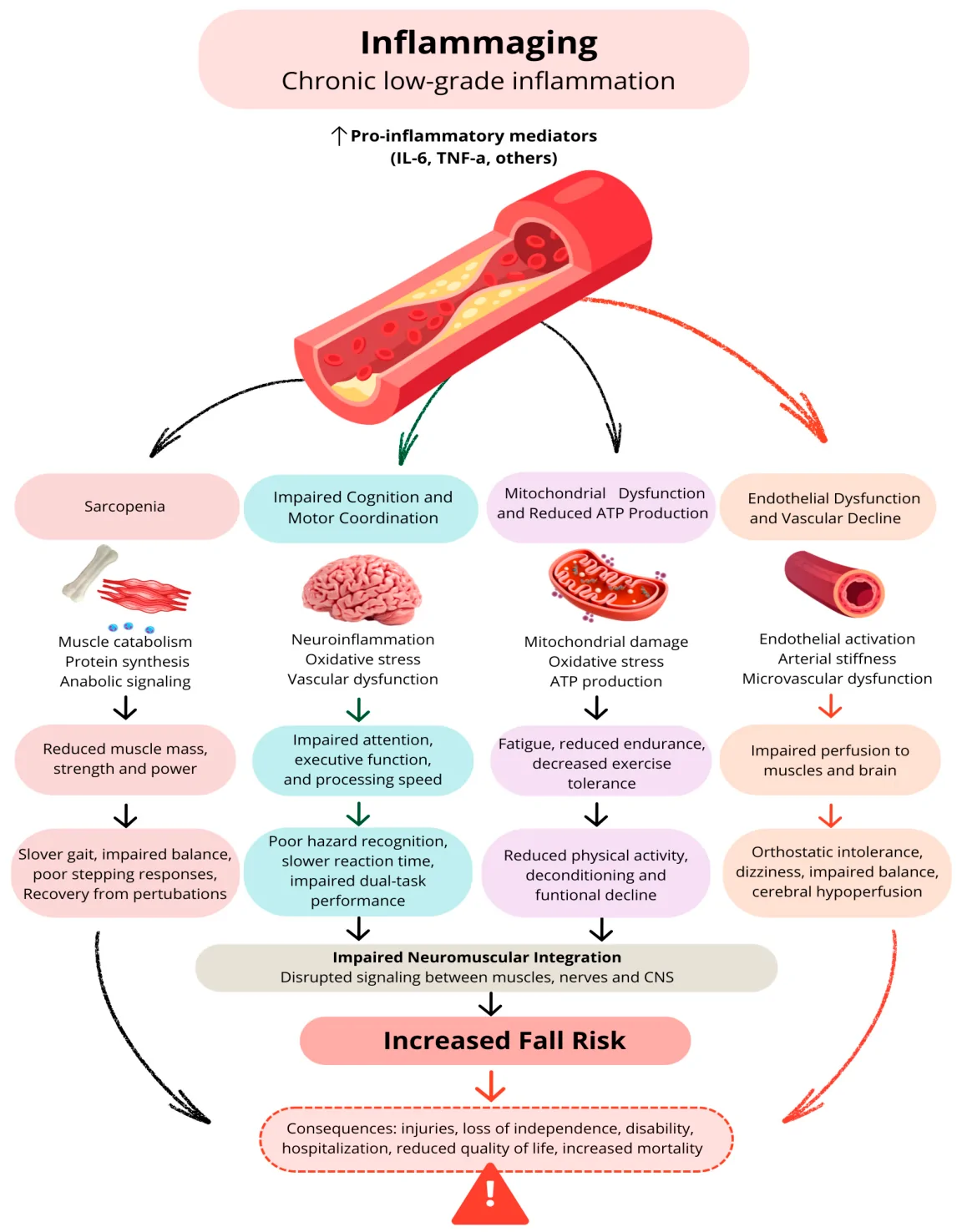
Biological pathways linking inflammaging, frailty, and fall risk in older women. The figure illustrates a conceptual framework whereby chronic low-grade inflammation promotes biological and functional impairments that increase fall susceptibility. The model is based on evidence synthesized from the reviewed literature and should be interpreted as a hypothesis-generating conceptual representation rather than a prospectively validated mechanistic model.

**Figure 2:**
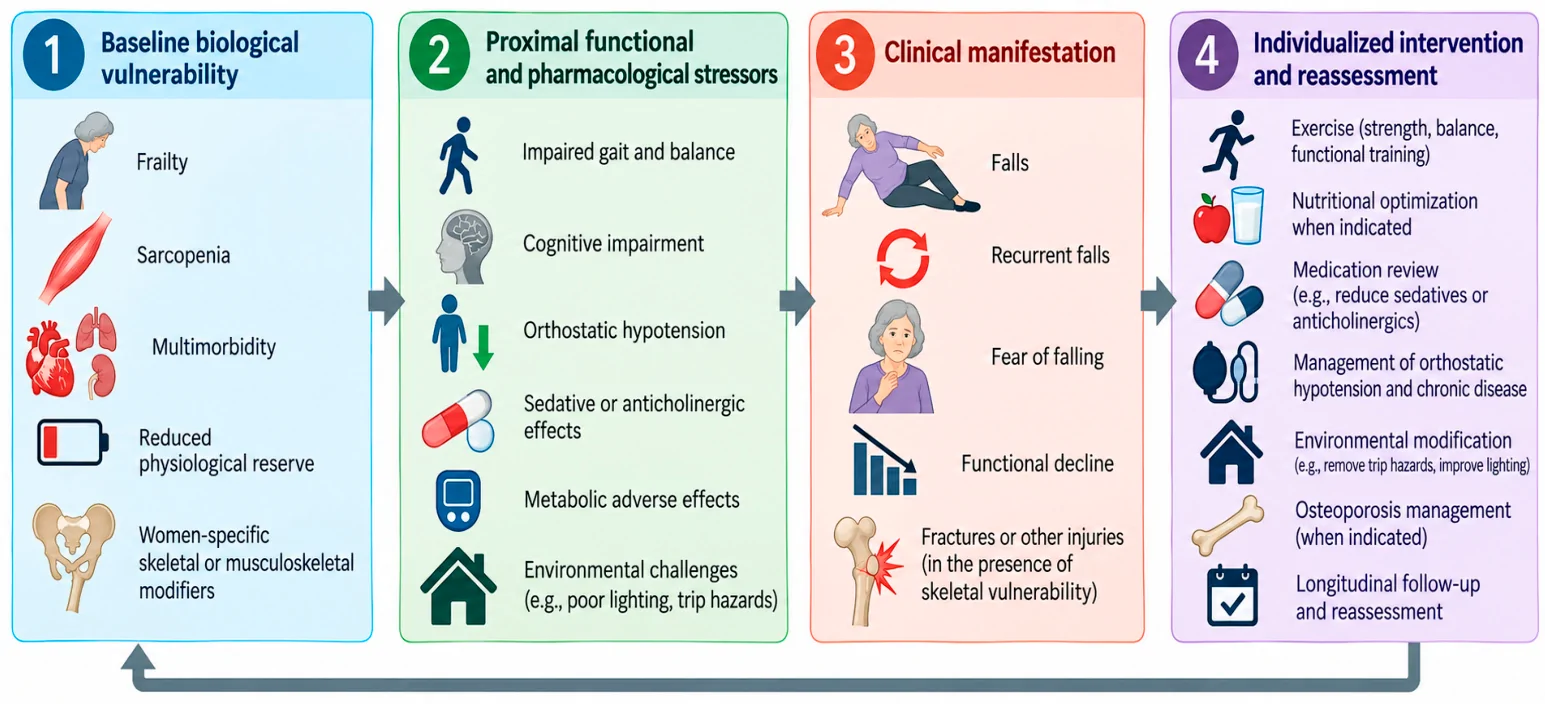
Proposed conceptual framework for fall-risk assessment and prevention in older women in primary care. The framework integrates baseline biological vulnerability, proximal functional and pharmacological stressors, fall-related outcomes and individualized multidomain interventions. It represents an evidence-informed, hypothesis-generating clinical model and has not been prospectively validated as a causal pathway or risk-prediction tool.

Elevated levels of inflammatory mediators, including interleukin-6, tumor necrosis factor-α, and C-reactive protein, have been associated with muscle catabolism, impaired regenerative capacity, reduced muscle strength and poorer physical performance [14,15,16]. The strongest evidence therefore links chronic inflammation to biological processes that may contribute indirectly to fall susceptibility—particularly frailty, sarcopenia, fatigue and impaired physical performance—rather than establishing inflammaging as an independent causal determinant of falls. Inflammatory activity has also been associated with cognitive decline and reduced functional capacity, which may further affect gait and balance [14,35]. However, these associations do not establish a direct causal pathway from systemic inflammation to incident falls and residual confounding by multimorbidity, physical inactivity, obesity, and underlying disease remains possible. Therefore, in the present review inflammaging is conceptualized as a contributor to biological vulnerability and loss of physiological reserve rather than as a stand-alone fall-risk factor. Figure 1 summarizes these proposed relationships. The pathways illustrated in Figure 2 should be interpreted as a hypothesis-generating synthesis of biologically plausible and observationally supported relationships rather than as a prospectively validated mechanistic cascade.

### 4.3. Metabolic Dysregulation and Energy Homeostasis

Age-related metabolic dysregulation may include insulin resistance, mitochondrial dysfunction, oxidative stress, altered substrate utilization, and impaired cellular energy production [21,35,50]. These abnormalities have been associated with reduced muscle quality, fatigue, impaired physical performance, and frailty [21,35,50]. The evidence linking these metabolic abnormalities directly to falls is considerably less developed than the evidence linking them to frailty and functional impairment. For this reason, metabolic dysregulation should primarily be considered an upstream contributor to biological vulnerability rather than an independently established determinant of incident falls. Metabolic disorders such as diabetes mellitus and obesity may further influence mobility through several pathways, including neuropathy, visual impairment, impaired muscle function, cardiovascular disease, pain, and reduced exercise capacity. In addition, treatment-related factors, including hypoglycaemia and orthostatic symptoms, may contribute to fall susceptibility independently of the metabolic disorder itself [21,24].

Accordingly, the proposed relationship between metabolic dysregulation and falls is likely to be mediated by intermediate phenotypes such as sarcopenia, frailty, fatigue, neuropathy and impaired physical performance rather than by a single direct mechanism [21,50].

### 4.4. Multimorbidity and Systemic Vulnerability

Multimorbidity is common in older adults and may increase fall susceptibility through the cumulative effects of chronic disease, functional impairment, sensory deficits, cognitive impairment, reduced exercise tolerance and treatment complexity [18,33,51]. Older women frequently live with combinations of osteoporosis, osteoarthritis, diabetes mellitus, cardiovascular disease, chronic pain, depression and other chronic conditions that may influence mobility and independence. However, the contribution of multimorbidity to fall risk depends not only on the number of diagnoses but also on disease severity, specific combinations of conditions, functional consequences and the treatments required to manage them. Multimorbidity may also increase exposure to polypharmacy and fall-risk-increasing medications [22,46,47]. This creates an important clinical interface between biological vulnerability and pharmacological burden, but the coexistence of multimorbidity, frailty, and polypharmacy should not be interpreted as evidence of a simple causal sequence [22,47]. These conditions frequently share underlying determinants and may influence each other bidirectionally [22,47].

From a primary-care perspective, the clinical significance of multimorbidity lies in its cumulative effect on physiological reserve and treatment complexity rather than in disease count alone. Assessment should therefore consider functional status, disease severity, medication burden, cognition, mobility, and previous falls rather than relying solely on the number of chronic diagnoses [18,24].

## 5. Pharmacological Determinants of Fall Risk

### 5.1. Polypharmacy and Medication Burden

Polypharmacy, commonly defined as the concurrent use of five or more medications, is highly prevalent among community-dwelling older adults and is consistently associated with an increased risk of adverse drug events, functional decline, and falls [35]. However, contemporary evidence suggests that the relationship between medication use and fall risk extends beyond the number of prescribed drugs. Medication burden, cumulative pharmacological exposure, and the use of fall-risk-increasing drugs (FRIDs) appear to be more clinically relevant determinants of fall susceptibility than medication count alone [36,37]. Older women are particularly vulnerable to medication-related adverse outcomes because multimorbidity frequently necessitates complex therapeutic regimens involving cardiovascular, psychotropic, analgesic, and osteoporosis-related medications. In addition, women have a longer life expectancy and a higher prevalence of chronic musculoskeletal disorders, depression, anxiety, and osteoporosis, all of which contribute to greater medication exposure [9,27]. Special attention should be paid to cumulative sedative and anticholinergic burden. Increased exposure to sedative medications impairs alertness, reaction time, gait, and postural stability, whereas anticholinergic medications may contribute to cognitive impairment, visual disturbances, dizziness, and impaired balance. Higher sedative and anticholinergic burden scores have consistently been associated with poorer physical performance, greater frailty severity, and an increased risk of falls in older adults [36]. Therefore, medication-related fall risk should be assessed using a comprehensive approach that considers medication burden, pharmacological profile, potential drug interactions, and individual patient characteristics rather than relying solely on conventional definitions of polypharmacy [37].

Polypharmacy commonly defined as the concurrent use of five or more medications is highly prevalent among older adults and has been associated with adverse drug events, functional decline, hospitalization and falls [46]. However, medication count alone provides an incomplete measure of pharmacological vulnerability. The clinical relevance of polypharmacy depends on the types of medications used, cumulative pharmacological burden, potential drug–drug and drug–disease interactions, dose, renal and hepatic function and the presence of fall-risk-increasing drugs (FRIDs) [12,24,48]. In older adults with multimorbidity, several medications may be clinically indicated and beneficial. Accordingly, polypharmacy should not automatically be equated with inappropriate prescribing [48]. The greater concern is inappropriate or high-risk polypharmacy, particularly when the medication regimen includes drugs that impair alertness, cognition, blood-pressure regulation, balance or neuromuscular function [12,24]. Cumulative sedative and anticholinergic burden is particularly relevant. Sedative medications may impair alertness, reaction time, psychomotor performance and postural stability, whereas anticholinergic medications may contribute to cognitive impairment, blurred vision, dizziness, urinary retention and impaired physical function [12,23]. Systematic evidence indicates that greater anticholinergic burden is associated with increased fall risk in older adults [23]. Medication-related fall susceptibility should therefore be assessed according to the pharmacological profile of the regimen rather than medication number alone. Structured review should consider FRIDs, sedative and anticholinergic burden, recent medication changes, dose escalation, drug interactions, renal function, orthostatic symptoms, and previous falls [12,24,48]. Although older women may experience substantial lifetime medication exposure because of greater longevity and multimorbidity, most evidence concerning medication burden and falls derives from mixed-sex older populations. These findings should therefore not be assumed to demonstrate a stronger medication-related effect specifically in women unless sex-stratified evidence is available.

### 5.2. Age-Related Pharmacokinetic and Pharmacodynamic Changes

Physiological aging alters both pharmacokinetic and pharmacodynamic responses to medications and may increase susceptibility to adverse drug reactions [10,11,46]. Age-related reductions in renal clearance and, for some drugs, hepatic metabolic capacity may increase systemic exposure and prolong elimination. Changes in body composition including reduced total body water and lean mass together with increased relative adiposity may alter the distribution of hydrophilic and lipophilic medications [10,11]. These pharmacokinetic changes are clinically relevant because drug accumulation may increase the likelihood of sedation, dizziness, hypotension, or cognitive impairment, particularly when renal function is reduced or multiple interacting medications are prescribed [10,11,46]. Pharmacodynamic changes may be equally important. Older adults may exhibit increased sensitivity to central nervous system-active medications and to drugs affecting cardiovascular homeostasis. These effects can manifest as sedation, impaired attention, psychomotor slowing, orthostatic hypotension, bradycardia and impaired postural responses [10,11,12]. Frailty may amplify these medication-related effects because reduced physiological reserve limits the ability to compensate for disturbances in blood pressure, cognition, alertness and balance [7,18]. However, evidence demonstrating a formal pharmacodynamic interaction between frailty and individual medication classes remains limited; this relationship should therefore be considered clinically plausible rather than definitively established. Renal function, hydration status, body composition, nutritional status, multimorbidity, and concurrent medication use should therefore be considered when evaluating medication-related fall susceptibility in older adults [12,24,46].

### 5.3. Fall-Risk-Increasing Drugs (FRIDs) and Specific Medication Classes

Several medication classes are associated with increased fall risk and are commonly grouped as FRIDs [12,24]. Current international fall guidelines recommend assessment of fall history before prescribing potential FRIDs and the use of structured tools during medication review [24].

Central nervous system-active medications

Benzodiazepines and non-benzodiazepine hypnotics (Z-drugs) may increase fall susceptibility through sedation, impaired attention, delayed reaction time, psychomotor impairment, and gait instability [12,24,26].

Antipsychotics, antidepressants, opioid analgesics, and selected antiepileptic or gabapentinoid agents may produce similar effects through combinations of sedation, dizziness, impaired coordination, and orthostatic symptoms [12,24,52]. The 2023 AGS Beers Criteria also identify several CNS-active drug classes as potentially inappropriate or requiring caution in older adults, particularly in those with a history of falls or fractures [26]. The magnitude of risk is influenced by dose, treatment duration, concomitant CNS-active medications, treatment initiation, dose escalation, and individual susceptibility. Therefore, class-level associations should not be interpreted as implying that every medication within a class confers the same risk to every patient.

Anticholinergic medications

Anticholinergic exposure deserves particular attention because cumulative burden may contribute to cognitive impairment, dizziness, blurred vision, reduced physical performance, and falls [23,26]. Rather than focusing only on individual strongly anticholinergic medications, clinicians should consider cumulative exposure across the entire regimen, because several drugs with moderate anticholinergic activity may together produce clinically relevant burden [23].

Cardiovascular medications and orthostatic hypotension

Antihypertensive agents, alpha-blockers, diuretics, and other cardiovascular drugs may contribute to fall susceptibility through excessive blood-pressure reduction, orthostatic hypotension, volume depletion, bradycardia, or electrolyte disturbances [13,24]. These medications should not be regarded as intrinsically inappropriate, because their cardiovascular benefits may be substantial. Clinical concern is greatest when hypotensive symptoms, recurrent falls, frailty, dehydration, recent dose escalation, or excessive blood-pressure lowering are present. Treatment decisions should therefore be individualized according to indication, treatment benefit, orthostatic measurements, and fall history [13,24].

Hyponatraemia and electrolyte disturbances

Medication-induced hyponatraemia represents an additional clinically relevant mechanism. Thiazide diuretics and several psychotropic drugs particularly serotonergic antidepressants may precipitate or aggravate hyponatraemia in susceptible older adults. Even mild chronic hyponatraemia has been associated with gait disturbance, attention impairment and increased fall susceptibility. Electrolyte abnormalities should therefore be considered when an older patient develops new dizziness, confusion, weakness or falls after initiation or dose modification of a potentially implicated medication [53].

Hypoglycaemia

Glucose-lowering medications capable of causing hypoglycemia may increase acute fall susceptibility through dizziness, weakness, visual disturbance, confusion and loss of consciousness [53]. This is particularly relevant in frail older adults or individuals with reduced oral intake, renal impairment or overly intensive glycemic treatment. The key point is not that all diabetes therapy increases fall risk, but that hypoglycaemia represents a treatment-related mechanism that should be considered during medication review.

Drugs affecting skeletal vulnerability

Medication-related risk should also be separated into drugs that increase the probability of falling and drugs that predominantly increase the probability of injury or fracture if a fall occurs [24,31].

Long-term systemic glucocorticoid exposure may reduce bone strength and increase fracture susceptibility [31]. Aromatase inhibitors used in postmenopausal breast cancer are also associated with accelerated bone loss and increased fracture risk. These skeletal effects should be distinguished from medication effects such as sedation or hypotension that directly increase fall propensity. Tamoxifen should not be grouped with aromatase inhibitors as uniformly detrimental to bone in postmenopausal women, because its skeletal effects differ and may include preservation of bone mineral density in this population. Long-term proton pump inhibitor exposure has also been associated in observational studies with fracture risk; however, residual confounding and uncertainty regarding causality should be acknowledged.

### 5.4. Medication Optimization and Deprescribing

Medication optimization is an important component of fall prevention in older adults particularly in individuals with polypharmacy, recurrent falls, frailty or exposure to FRIDs [24]. Current global fall guidelines recommend a structured medication review preferably using a validated tool and consideration of deprescribing psychotropic, cardiovascular and other FRIDs as part of a multidomain fall-prevention strategy. Structured prescribing tools such as STOPP/START version 3 can help identify potentially inappropriate medications and prescribing omissions [25]. The 2023 AGS Beers Criteria provide an additional framework for identifying medications that may be inappropriate or require caution in adults aged 65 years and older, although the criteria should support rather than replace individualized clinical decision-making [26]. Medication review should include confirmation of the current indication, expected benefit, dose, duration of therapy, renal and hepatic function, adverse effects, relevant drug–drug and drug–disease interactions, cumulative anticholinergic or sedative burden, and the patient’s treatment goals and preferences [23,24,25,26,48,49]. Importantly, the evidence does not support the assumption that deprescribing FRIDs alone consistently reduces falls. A systematic review and meta-analysis of FRID withdrawal trials found little to no difference in fall rate or fall incidence when deprescribing was used as a sole fall-reduction strategy. A broader systematic review of medication reviews and deprescribing interventions similarly found uncertainty regarding effectiveness when these interventions were delivered alone.

Medication optimization should therefore be incorporated into multidomain prevention rather than presented as an independently proven fall-prevention intervention [24,49]. Such programs may combine medication review with exercise, gait and balance assessment, management of orthostatic hypotension, vision assessment, environmental modification, nutritional assessment, and management of frailty and chronic disease [24,27,28,29,30]. Deprescribing should also not mean indiscriminate withdrawal. Decisions should be individualized and based on expected benefit, potential harm, life expectancy where relevant, frailty, treatment priorities and patient preferences [25,48,49]. Where medication discontinuation may cause withdrawal symptoms or disease destabilization, dose reduction and follow-up should be planned carefully [49]. For older women, medication review should additionally consider therapies that influence skeletal health, because pharmacological risk may involve not only the probability of falling but also the probability of fracture following a fall.

## 6. Integrating Biological Vulnerability and Pharmacological Burden: A Conceptual Framework for Fall Risk

Falls in older women should not be understood as the consequence of a single biological abnormality or medication exposure. Rather fall susceptibility reflects the cumulative influence of age-related physiological changes, frailty, sarcopenia, multimorbidity, functional impairment, medication exposure, and environmental or behavioral factors [18,24]. The individual relationships among these determinants are already well established. The purpose of the present framework is therefore not to propose new individual risk factors, but to organize existing biological, functional and pharmacological evidence into an integrated clinical model relevant to older women in primary care. Frailty provides a clinically useful representation of reduced physiological reserve [17,18]. Sarcopenia, impaired physical performance, cognitive impairment, orthostatic hypotension, multimorbidity and nutritional deficits may further reduce the capacity to maintain functional stability during physiological or environmental stress [18,20,24,36,37].

Within this framework, biological vulnerability is considered a background state that may modify the clinical consequences of pharmacological exposure. This does not imply that frailty and medications interact synergistically in a formally demonstrated causal sense. Rather a medication-related perturbation—such as sedation, orthostatic hypotension, bradycardia, cognitive impairment, electrolyte disturbance or hypoglycemia—may have greater functional consequences in an individual with limited physiological reserve [12,18,24]. For example, a modest reduction in blood pressure or alertness may be tolerated without clinical consequences in a robust older adult, whereas the same disturbance may contribute to instability in an individual with impaired balance, muscle weakness or previous falls. This concept is consistent with the broader geriatric principle that adverse outcomes often emerge when multiple modest impairments accumulate beyond an individual’s compensatory capacity. However, the magnitude of interaction between frailty and specific medication classes remains incompletely defined and requires prospective investigation [18,35,54].

Functional vulnerability as the clinical interface

The concept of functional vulnerability provides a useful clinical bridge between biological reserve and pharmacological burden. Functional vulnerability reflects the cumulative effect of impaired gait, reduced muscle strength, balance deficits, cognitive impairment, orthostatic symptoms, sensory deficits, and medication-related adverse effects on an individual’s ability to maintain stability during everyday activities [18,24,36,37]. In the present framework, functional vulnerability is not proposed as a newly validated diagnostic construct or risk score. Rather, it is used descriptively to integrate multiple clinically observable domains that may jointly influence fall susceptibility. As physiological reserve declines, relatively minor stressors—including acute illness, dehydration, environmental hazards, medication initiation or dose escalation, and transient metabolic disturbances—may become clinically important. The framework therefore emphasizes threshold effects and accumulation of vulnerability rather than assuming a simple linear relationship between the number of risk factors and fall probability.

Primary-care application of the framework

From a primary-care perspective, fall-risk assessment should extend beyond the identification of isolated risk factors. Current international guidance supports multidomain assessment incorporating fall history, gait and balance, medication use, cardiovascular factors, cognition, vision, functional status, and other relevant clinical determinants [24]. For older women, this assessment may additionally require attention to osteoporosis, fracture history, postmenopausal skeletal vulnerability, sarcopenia, and therapies that affect bone health [5,6,31,32,43].

These factors may influence either the probability of falling or the probability of serious injury following a fall and should therefore be considered separately. A pragmatic primary-care assessment may include: previous falls and fall-related injuries; frailty status and physical performance; gait, balance, and muscle strength; orthostatic blood pressure and cardiovascular symptoms; cognition and vision; multimorbidity and nutritional status; medication burden, with particular attention to FRIDs; sedative and anticholinergic burden; recent medication initiation or dose changes; osteoporosis; and fracture risk. The objective is not to calculate a novel numerical risk score but to identify potentially modifiable contributors and determine where multidomain intervention may be appropriate.

Accordingly, the proposed framework should be viewed as an evidence-informed structure for organizing clinical assessment and intervention rather than as a validated causal model or prediction tool. Prospective studies are required to determine whether integrating frailty, functional performance, medication burden, and women-specific modifiers improves fall-risk prediction or clinical outcomes beyond established multidomain assessment approaches.

## 7. Clinical Implications for Primary Care

Effective fall prevention in older women requires a multidomain assessment that considers frailty, physical performance, medication burden, multimorbidity, cognition, nutrition, previous falls and environmental or behavioral factors [24,28,55]. Because comprehensive geriatric assessment may not always be feasible during a routine primary-care consultation, a pragmatic stepwise approach is preferable. Initial screening should identify individuals who require more detailed assessment rather than attempting to perform a full multidomain evaluation in every older patient [24,55,56,57].

### 7.1. Initial Risk Identification

Initial assessment may include previous falls, fear of falling, mobility limitations, orthostatic symptoms, medication exposure, cognition, and frailty status [38,47,48]. Validated frailty instruments such as the Clinical Frailty Scale (CFS) or FRAIL questionnaire may be used to support case findings [47,48]. These tools should be regarded as screening instruments rather than stand-alone diagnostic or fall-prediction tools. Their results should be interpreted together with functional performance, clinical history and comorbidity burden. Individuals identified as vulnerable may benefit from further assessment of: gait speed; Timed Up and Go test; chair-stand performance; grip strength; balance; activities of daily living; instrumental activities of daily living; cognition; orthostatic blood pressure; visual function; nutritional status; and medication burden. The objective is to identify potentially modifiable contributors to fall susceptibility rather than to generate a single numerical risk score [24,42].

### 7.2. Exercise and Physical Function

Exercise remains one of the most consistently supported interventions for reducing falls in older adults [38,50,51,52,53,58]. Programs that combine balance training, progressive resistance exercise, gait practice, and functional task training are generally more relevant to fall prevention than isolated strength or aerobic exercise alone. In frail and prefrail older adults, exercise may improve muscle strength, mobility, balance, and confidence [24,27,28,29,30,59]. However, exercise prescriptions should be individualized according to baseline function, comorbidity, fall history, and supervision needs. Frail individuals may require lower starting intensity, closer monitoring, and gradual progression. For older women, attention should also be given to osteoporotic fracture risk when planning higher-load or impact-based activity [27,28].

### 7.3. Nutrition and Sarcopenia Management

Nutritional assessment is particularly relevant when weight loss, low protein intake, sarcopenia, or undernutrition is suspected [28,39,40]. Adequate energy and protein intake should support muscle maintenance, but supplementation should be individualized rather than routinely prescribed to all older women at risk of falls. Vitamin D deficiency should be corrected when documented or when supplementation is otherwise clinically indicated [38,60]. The primary-care goal is therefore not universal supplementation, but identification and correction of clinically relevant nutritional deficits that may worsen frailty, sarcopenia, or physical performance [24,40].

### 7.4. Medication Review

Medication review should be incorporated into fall-risk assessment, particularly in individuals with recurrent falls, frailty, polypharmacy, or exposure to FRIDs [24,25]. Structured tools such as STOPP/START may assist in identifying potentially inappropriate prescribing [25]. Medication review should include more than medication count. Clinicians should assess indication, dose, duration, renal function, recent medication changes, sedative burden, anticholinergic burden, orthostatic symptoms, hypoglycaemia risk, electrolyte disturbances, and potential drug–drug or drug–disease interactions. Deprescribing should be individualized and should not be considered synonymous with automatic medication withdrawal. The aim is treatment optimization, balancing expected benefit against potential harm and the patient’s functional status and preferences.

Where possible, medication review should be integrated into a broader multidomain intervention rather than used as a stand-alone fall-prevention measure [24,49].

### 7.5. Orthostatic and Cardiovascular Assessment

Orthostatic blood pressure measurement should be considered in older adults with dizziness, syncope, recurrent falls, recent medication changes, or antihypertensive treatment. Potential contributors include dehydration, excessive blood-pressure lowering, bradycardia, and medication-related autonomic effects. Management should focus on identifying the cause and modifying reversible factors while preserving appropriate cardiovascular treatment where benefit remains clear. This distinction is important because cardiovascular medications should not be indiscriminately reduced solely because a patient has fallen.

### 7.6. Bone Health and Fracture Prevention

Because fall occurrence and fracture occurrence are not equivalent outcomes, bone health should be assessed separately from fall propensity. In older women, osteoporosis, previous fragility fracture, low bone mineral density, glucocorticoid exposure, and therapies associated with skeletal loss may increase the likelihood that a fall results in serious injury [13,24]. Accordingly, fall prevention and fracture prevention should proceed in parallel: one aims to reduce the probability of falling, while the other aims to reduce the consequences of a fall.

### 7.7. Multidisciplinary and Coordinated Care

Optimal management may require collaboration among primary care physicians, geriatricians, pharmacists, physiotherapists, nurses, dietitians, and other allied health professionals [38,56,57]. However, multidisciplinary care should be targeted to patient need and local availability rather than presented as a mandatory model for every individual. Primary care remains well positioned to coordinate: frailty screening; medication review; referral for exercise or rehabilitation; nutritional assessment; osteoporosis management; evaluation of cognition and vision; environmental risk modification; and follow-up after falls [61]. Longitudinal reassessment is essential because frailty status, medication exposure, and fall risk may change over time [5,31,32,43].

Population aging is accelerating worldwide, and women constitute a substantial proportion of the oldest-old population because of their greater life expectancy [44,62,63]. Consequently, healthcare systems are likely to face an increasing burden of falls, fall-related injuries, fractures, disability and loss of independence among older women [64]. Despite the large body of literature on falls in older adults, several important knowledge gaps remain. These include limited standardization of frailty assessment, insufficient representation of women-specific analyses, heterogeneity in the definition and measurement of polypharmacy and medication burden, and the predominance of observational evidence for several proposed biological pathways [8,22,65,66].

## 8. Challenges and Future Directions

### 8.1. Need for Better Women-Specific Evidence

A major limitation of the current evidence base is that many studies relevant to frailty, inflammaging, sarcopenia, medication burden, and falls include mixed-sex populations without reporting sex-stratified outcomes [8,9,22].

Although postmenopausal hormonal changes, osteoporosis, sarcopenia, multimorbidity, and medication exposure are clinically relevant to older women, these factors are frequently investigated separately rather than within integrated models [5,6,31,32,44]. Future studies should therefore include adequately powered analyses in older women and should report sex-stratified outcomes where mixed-sex cohorts are used. This would help determine whether observed associations differ in magnitude, mechanism, or clinical consequence between women and men rather than assuming that findings from general geriatric populations apply equally to both sexes. This change directly addresses the key reviewer concern about extrapolation from mixed-sex populations.

### 8.2. Prospective Validation of the Proposed Framework

The proposed framework integrates frailty, biological vulnerability, functional impairment, medication burden, and women-specific modifiers. However, the framework has not been prospectively validated and should not be interpreted as a prediction model. Future research should therefore evaluate whether combinations of frailty status, gait and balance measures, muscle strength, previous falls, cognition, orthostatic blood pressure, multimorbidity, medication burden, FRID exposure, nutritional status and bone-health variables improve prediction of falls or fall-related injuries beyond existing multidomain assessment approaches. Prospective cohort studies should evaluate not only first falls but also recurrent falls, fractures, hospitalization, functional decline, institutionalization, and loss of independence. This is more specific than the original statement that “novel risk-stratification algorithms may improve identification” [24].

### 8.3. Frailty, Inflammation, and Mechanistic Research

The biological relationships among chronic inflammation, sarcopenia, metabolic dysfunction, and frailty remain clinically relevant but incompletely understood [14,15,16,21,50]. Future mechanistic studies should distinguish biomarkers associated with frailty from biomarkers that independently predict falls. This distinction is important because elevated inflammatory markers may reflect chronic disease burden, obesity, reduced physical activity, sarcopenia, infection or other age-related conditions. Longitudinal studies are needed to determine whether inflammatory or metabolic biomarkers provide clinically useful information beyond established functional measures such as gait speed, muscle strength, balance, and previous fall history [19,20,24]. This prevents the Future Directions section from assuming biomarkers will improve clinical prediction.

### 8.4. Pharmacological Research

The interaction between frailty and medication exposure remains another important research priority.

Future pharmacological studies should move beyond medication count and examine specific drug classes, cumulative sedative and anticholinergic burden, dose, treatment initiation, dose escalation, renal function, drug–drug interactions and clinically relevant adverse effects such as orthostatic hypotension, hyponatremia, hypoglycemia, cognitive impairment and gait disturbance. Frailty-stratified analyses are particularly needed to determine whether the same medication exposure has different functional consequences in robust, prefrail and frail older adults. Randomized studies should also evaluate which medication-optimization strategies are most effective when incorporated into multidomain fall-prevention programs. Importantly, future trials should distinguish between reducing medication burden, reducing FRID exposure, and improving prescribing appropriateness, because these are related but not equivalent interventions [25,48,49].

### 8.5. Implementation in Primary Care

An important challenge is translating evidence into routine practice. Barriers include limited consultation time, inconsistent use of frailty assessment, fragmented healthcare pathways, insufficient access to rehabilitation, variable pharmacist involvement and limited integration of fall-risk assessment into electronic health records [62,67]. Implementation research should therefore focus not only on whether an intervention is effective, but also on whether it is feasible, sustainable, acceptable to patients and clinicians, and adaptable to different primary-care systems. Attention should be given to pragmatic approaches that can be implemented during routine consultations, including brief frailty screening, structured medication review, orthostatic blood-pressure assessment, fall-history documentation, targeted referral for physical therapy, nutritional assessment and osteoporosis management [24,66,68].

### 8.6. Digital Health and Artificial Intelligence

Emerging digital technologies, including wearable sensors, remote monitoring, electronic health record-integrated screening, and artificial intelligence-assisted decision support, may provide additional opportunities for detecting functional decline or changes in fall-related risk [69,70,71]. However, their clinical utility should not be assumed solely from technical performance. Future studies should evaluate external validation; usability; false-positive rates; patient acceptability; digital exclusion; cost-effectiveness; workflow integration; and clinically meaningful outcomes. Digital tools should therefore be considered potential adjuncts to clinical assessment rather than replacements for functional evaluation, medication review, or clinical judgment.

This is much safer than presenting AI and wearables as an inevitable next step.

### 8.7. Priority Research Agenda

**Figure 3:**
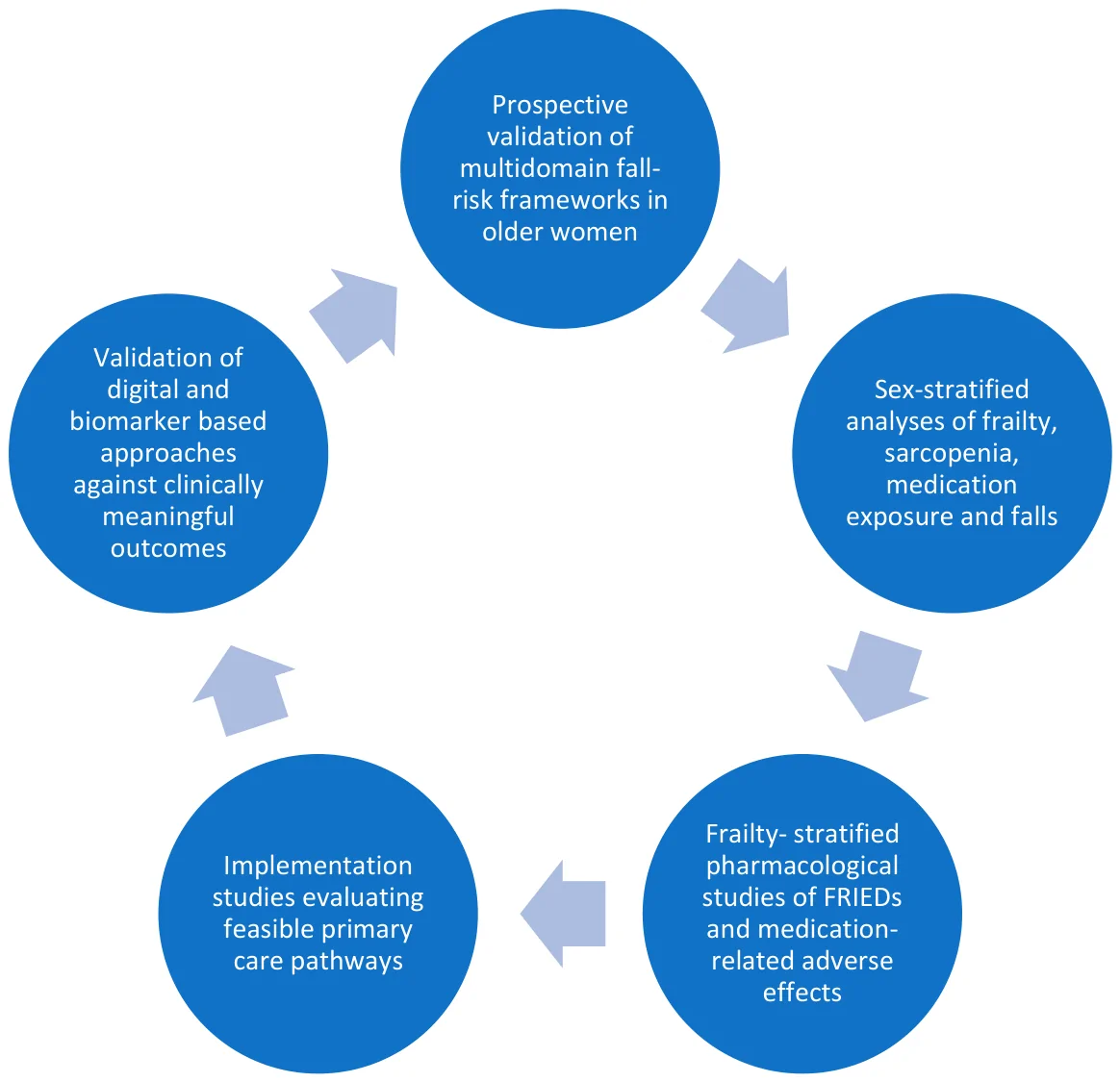
Priority research agenda for fall risk in older women.

Future research should prioritize five areas:

Based on the evidence gaps identified in this narrative review, five priority areas for future research are summarized in Figure 3. These priorities may help determine which components of the proposed framework add clinically useful information beyond existing fall-prevention approaches and which are primarily explanatory rather than predictive. Future research should determine whether integration of frailty, functional performance, medication burden, women-specific modifiers, and selected biological markers improves prediction or prevention of clinically meaningful outcomes beyond established multidomain fall assessment. Until such models are prospectively validated, the primary clinical priority should remain systematic identification of modifiable functional, pharmacological, and skeletal risk factors within feasible primary-care pathways.

**Limitations of the Review.** This review has several methodological limitations that should be acknowledged. First, its narrative design structure does not provide the same level of reproducibility or methodological control as a formal systematic review. Second, no formal risk-of-bias assessment or certainty-of-evidence grading was performed. Third, the included evidence was heterogeneous with respect to study populations, definitions of frailty and polypharmacy, medication exposures, outcomes and study designs, which limits direct comparison across studies. Fourth, a substantial proportion of the available evidence was derived from mixed-sex or general older populations rather than exclusively from older women and findings from such studies may not be fully generalizable to women specifically. Fifth, the review was restricted to publications available in English, introducing the possibility of language bias. Finally, despite the use of predefined thematic domains and independent screening by two reviewers, qualitative selection, interpretation, and synthesis of the evidence remain partly dependent on reviewer judgment and may therefore be subject to residual subjectivity. These limitations should be considered when interpreting the proposed conceptual framework, which is intended as an evidence-informed, hypothesis-generating model rather than a validated causal or predictive tool.

## 9. Conclusions

Falls in older women are multifactorial events arising from the combined influence of biological vulnerability, functional impairment, multimorbidity, and medication-related factors. Frailty and sarcopenia are among the most clinically relevant markers of reduced physiological reserve and impaired mobility, whereas inflammaging and metabolic dysregulation appear to contribute mainly through their associations with frailty, muscle dysfunction, and functional decline rather than as independently established causes of falls. Medication-related risk should likewise be interpreted according to specific pharmacological mechanisms rather than medication count alone. Fall-risk-increasing drugs may contribute through sedation, cognitive impairment, orthostatic hypotension, bradycardia, electrolyte disturbances, hypoglycaemia, and gait instability, whereas other therapies may predominantly modify fracture susceptibility through adverse skeletal effects. The principal contribution of this review is therefore not the identification of new individual risk factors, but the integration of established biological, functional, pharmacological, and women-specific determinants into a clinically oriented primary-care framework. From a practical perspective, fall prevention should combine early recognition of frailty and functional decline, assessment of previous falls and gait or balance impairment, individualized medication review, evaluation of orthostatic and metabolic adverse effects, nutritional assessment, exercise-based interventions, and parallel consideration of fracture risk and bone health. Medication optimization should be incorporated into multidomain fall prevention rather than considered a stand-alone intervention, and women-specific skeletal vulnerability should be assessed separately from factors that directly increase the probability of falling. Because much of the available evidence derives from observational studies and mixed-sex older populations, the proposed framework should be regarded as an evidence-informed, hypothesis-generating model rather than as a validated causal or predictive tool. Prospective studies are needed to determine whether integrating frailty, functional performance, medication burden, and women-specific modifiers improves prediction of falls, recurrent falls, fractures, functional decline, and other clinically meaningful outcomes beyond existing multidomain assessment approaches.

## Figures and Tables

**Table 1:** Representing key evidence informing the biological, pharmacological and clinical domains of the narrative synthesis.

Reference	Study Type/Population	Sex Specificity	Main Domain	Principal Finding Relevant to This Review	Role in the Present Synthesis
Fried et al. [17]	Prospective cohort of older adults	Mixed sex	Frailty	Established the physical frailty phenotype and its relationship with adverse health outcomes	Foundational definition of frailty as reduced physiological reserve
Gordon et al. [8]	Systematic review and meta-analysis	Sex-specific comparison	Frailty/sex	Frailty prevalence was generally higher in women than in men	Supports the rationale for considering sex-specific vulnerability
Hoogendijk et al. [18]	Clinical/public-health review	Mixed sex	Frailty	Frailty is associated with impaired resilience and multiple adverse outcomes in older adults	Supports interpretation of frailty as a background state of biological vulnerability
Kojima [19]	Systematic review and meta-analysis of community-dwelling older adults	Predominantly mixed sex	Frailty/falls	Frailty was associated with an increased risk of future falls	Provides relatively direct evidence linking frailty with fall susceptibility
Yeung et al. [20]	Systematic review and meta-analysis of older adults	Mixed sex	Sarcopenia/falls/fractures	Sarcopenia was associated with greater risk of falls and fractures	Strengthens the direct evidence connecting sarcopenia with fall-related outcomes
Cruz-Jentoft et al. [4]	European consensus statement	Mixed sex	Sarcopenia	Emphasized low muscle strength as a key characteristic of probable sarcopenia and physical performance as a marker of severity	Supports emphasis on muscle strength and function rather than muscle mass alone
Ferrucci & Fabbri [14]; Franceschi et al. [16]	Narrative/state-of-the-art reviews	Mixed sex/general aging	Inflammaging	Chronic low-grade inflammation is linked to frailty, muscle catabolism, and age-related functional decline	Supports inflammaging as an upstream contributor to vulnerability, not as a proven independent cause of falls
Sun et al. [21]	Prospective multicohort study	Mixed sex	Metabolic dysregulation/frailty	Metabolic abnormalities were associated with frailty across prospective cohorts	Supports an indirect metabolic pathway through frailty; does not establish a direct effect on falls
Gutiérrez-Valencia et al. [22]	Systematic review	Mixed-sex older adults	Frailty/polypharmacy	Frailty and polypharmacy were consistently associated, although the evidence was heterogeneous and largely observational	Supports a potentially bidirectional relationship while avoiding causal claims
Stewart et al. [23]	Systematic prognostic review	Mixed-sex older adults	Anticholinergic burden/falls	Greater anticholinergic burden was associated with increased fall risk	Supports assessment of cumulative anticholinergic exposure rather than medication count alone
Seppala et al. [12]	EuGMS expert position paper	Mixed-sex older adults	FRIDs	Identified important medication classes and mechanisms contributing to medication-related fall susceptibility	Supports structured evaluation of fall-risk-increasing drugs
Montero-Odasso et al. [24]	International clinical practice guideline	Mixed-sex older adults	Falls prevention	Recommends multidomain fall-risk assessment, including medication review and targeted management of modifiable factors	Provides the principal clinical framework for primary-care recommendations
O’Mahony et al. [25]	Expert consensus/prescribing criteria	Mixed-sex older adults	Potentially inappropriate prescribing	STOPP/START version 3 provides criteria for identifying potentially inappropriate medications and prescribing omissions	Supports structured medication optimization in older adults
2023 AGS Beers Criteria Update Expert Panel [26].	Expert consensus criteria	Mixed-sex adults ≥65 years	Medication safety/FRIDs	Identifies medication classes requiring avoidance or caution in older adults, including patients with a history of falls or fractures	Supports discussion of CNS-active and anticholinergic medications
Apóstolo et al. [27]	Systematic review	Mixed-sex older adults	Prefrailty/frailty intervention	Multicomponent interventions, particularly exercise-based strategies, may improve frailty-related outcomes	Supports describing prefrailty as potentially modifiable, rather than universally reversible
Dent et al. [28]	International clinical practice guideline	Mixed-sex older adults	Frailty management	Supports individualized multidomain management, with exercise as a major intervention and nutrition according to clinical need	Supports revised exercise and nutritional recommendations
Lewis et al. [29]/Morello et al. [30]	Systematic reviews/meta-analyses	Mixed-sex older adults	Fall prevention	Multifactorial or multidomain approaches can address several modifiable fall-risk domains	Supports integrated rather than single-component prevention
Compston et al. [31]/Greco et al. [32]	Clinical review(s)	General/mixed sex	Osteoporosis/skeletal vulnerability	Osteoporosis and sarcopenia contribute differently to adverse outcomes in older	

## Data Availability

No new data were created or analyzed in this study. Data sharing is not applicable to this article.

## Abbreviations

The following abbreviations are used in this manuscript:

ADLs	Activities of Daily Living
ADR(s)	Adverse Drug Reaction(s)
CFS	Clinical Frailty Scale
CGA	Comprehensive Geriatric Assessment
CNS	Central Nervous System
CRP	C-Reactive Protein
FRIDs	Fall-Risk-Increasing Drugs
IADLs	Instrumental Activities of Daily Living
IL-6	Interleukin-6
MEDLINE	Medical Literature Analysis and Retrieval System Online
PK	Pharmacokinetics
PD	Pharmacodynamics
PPI(s)	Proton Pump Inhibitor(s)
STOPP	Screening Tool of Older Persons’ Prescriptions
START	Screening Tool to Alert to Right Treatment
TNF-α	Tumor Necrosis Factor Alpha
TUG	Timed Up and Go test
